# T-helper-associated cytokines expression by peripheral blood mononuclear cells in patients with polypoidal choroidal vasculopathy and age-related macular degeneration

**DOI:** 10.1186/s12886-016-0251-z

**Published:** 2016-06-07

**Authors:** Ying Yu, Xiang Rong Ren, Feng Wen, Hui Chen, Shao Bo Su

**Affiliations:** State Key Laboratory of Ophthalmology, Zhongshan Ophthalmic Center, Sun Yat-sen University, 54 South Xianlie Road, Guangzhou, 510060 China

**Keywords:** T-helper cell, Cytokine, Peripheral blood mononuclear cell, Age-related macular degeneration, Polypoidal choroidal vasculopath

## Abstract

**Background:**

Immune responses play a key role in the pathogenesis and progression of polypoidal choroidal vasculopath (PCV) and age-related macular degeneration (AMD). In this study, we determined the Th cell-associated immune responses by measuring the cytokine expression of peripheral blood mononuclear cells (PBMC) in both PCV and neovascular AMD (nAMD) patients.

**Methods:**

Twenty-seven nAMD patients, 33 PCV patients and a gender- and age-matched group of 18 healthy individuals were involved in this study. The Th-cell cytokine profiles including levels of interferon-gamma (INF-γ), interleukin (IL)-17A and IL-4 in cultures of PBMCs were determined by enzyme-linked immunosorbent assay (ELISA).

**Results:**

IFN-γ,IL-17A and IL-4 production was significantly increased after stimulation with PHA. The levels of IFN-γ and IL-4 in PHA-stimulated cultures were higher in PCV and nAMD patients than that in healthy controls (*P* = 0.038,*P* = 0.014), while no difference was found between PCV and nAMD (all *P* > 0.05). No significant difference in IL-17A level in PHA-stimulated cultures was found among PCV, nAMD and control groups (*P* > 0.05).

**Conclusions:**

These findings suggest that circulating IFN-γ and IL-4 producing Th1 and Th2 cells may involve in the pathogenesis of PCV and nAMD. PCV may have the similar immune responses with nAMD.

## Background

Age-related macular degeneration (AMD) is a leading cause of irreversible visual disability and blindness among elderly in developed countries [1]. An advanced stage of AMD is characterized by choroidal neovascularization (neovascular AMD, nAMD) or geographic atrophy. Polypoidal choroidal vasculopath (PCV) exhibits commonalities with nAMD in that both are choroidal vasculopathy associated with subretinal hemorrhage, scars and fibrosis [2]. However, PCV is characterized by inner choroidal vascular networks ending in polypoidal structures at the borders of the lesion, while nAMD is characterized by choroidal neovascularization [3]. PCV still remains a subject of debate as to whether it is a subtype of AMD or its own distinct entity. The differences between the pathogeneses of PCV and nAMD are still unknown.

Recent information supports the notion that immune mechanisms play an important and perhaps central role in the pathogenesis of AMD [4]. CD4^+^ T cells are key players in immune responses. Among the T-helper cells, each subset produces different effector cytokines. Th1 cells predominantly secrete Interferon-gamma (IFN-γ), Th2 cells primarily produce IL-4 [5], CD4^+^ Th cells also produce IL-17, denoted as Th17 cells [6]. It is suggests that the underlying mechanism leading to AMD is the decline of the ocular down-regulatory immune environment [7]. The subsequent activation of the immune system would lead to T-cell sensitization. The accumulation of complement component 5a (C5a) and C3a in drusen promote choroidal neovascularization (CNV), which is the hallmark of wet AMD [8]. C5a promotes interleukin (IL)-22 and IL-17 expression by human CD4^+^ T cells [9]. IL-17A was initially reported to be mainly expressed by activated CD4^+^ T cells. It was implicated in the pathogenesis of various autoimmune diseases including uveitis, arthritis, multiple sclerosis and inflammatory bowel disease [10]. IL-17A rs2275913G/A and rs3748067C/T polymorphisms are associated with increased risk of AMD [11]. IFN-γ, a soluble cytokine associated with innate and adaptive immunity, is considered to be a pro-inflammatory factor. Constituents of drusen such as amyloid beta and advanced glycation endproducts (AGE) are capable of activating the IFN-γ pathway [12]. IFN-γ promotes proinflammatory responses by activating proinflammatory cytokines and chemokines, thereby recruiting immune cells such as macrophages, microglia, NK and T cells into inflammatory sites [13, 14]. IFN-γ induces a dose- and time-dependent increase in complement factor B mRNA expression in primary macrophages and macrophage cell lines [15] and increases CXCL11 mRNA expression and CXCL11 protein secretion by ARPE-19 cells [16]. IFN-γ also induces VEGF expression in human retinal pigment epithelial cells. Unexpectedly, IFNγ-induced VEGF secretion in human retinal pigment epithelial cells (RPE) was not through the classic IFN-γ Jak/Stat1 pathway, but through the PI-3 K/mTOR/p70-S6K-translational regulation pathway [17]. In laser-induced CNV model, NK cell depletion leads to decreased areas of CNV, macrophage infiltration and reduced mRNA expression of VEGFs and IFN-γ in the choroid, suggesting that IFN-γ-secreting NK cells promote angiogenesis by enhancing VEGF expression via macrophage activation [18]. IFN-γ may involve in AMD pathogenesis through macrophage polarization. INF-γ can selectively promote polarization into M1 subtype [19]. M1 subtype is predominantly proinflammatory and there is a pathological shift towards M1 subtype with the development of AMD [13].

Although IFN-γ and IL-17 are shown to be the major product of inflammasome cascade activation, few study focus on the immune-inflammatory response of PCV. The Th-cytokine levels of PCV and nAMD in peripheral blood mononuclear cells (PBMCs) have not been previously studied. In the present study, we studied the production of IFN-γ, IL-17A, and IL-4 in cultures of PBMCs from PCV and nAMD patients.

## Methods

### Subjects and sample preparation

Twenty-seven nAMD patients, 33 PCV patients and a gender- and age-matched group of 18 healthy individuals were selected from the Outpatient Clinics at the Zhongshan Ophthalmic Centre for the study. Informed consent was obtained from all patients and controls before entering the study. Research was performed in accordance with the ethical standards of the Declaration of Helsinki and the internal Ethics Committees of Sun Yat-sen University, Guangzhou, China, which approved all of the protocols.

Only patients without infectious diseases and not using immunosuppressive drugs were enrolled in the study. Moreover, patients with other neovascularized maculopathies, such as pathologic myopia, angioid streaks, and retinal angiomatous proliferation were excluded. Patients with media opacities preventing clear visualization of the macula were excluded from the study. Patients received intravitreal anti-vascular endothelial growth factor (VEGF) therapy were also excluded from the study.

The diagnosis of PCV was based on identification of characteristic polypoidal choroidal vascular dilations with or without branching inner choroidal vessels on ICGA. All PCV patients enrolled in this study met the criteria of definitive cases of PCV, as proposed by the Japanese Study Group of PCV [20]. Cases diagnosed as probable were excluded because they were difficult to distinguish from AMD cases. The diagnosis of nAMD was based on identification of choroidal neovascularization by fluorescein angiography and ICGA. All nAMD cases were classified as stage 4 by the Rotterdam Study classification [21].

A whole-blood (12 ml) sample was taken from each study participant and placed in a sterile tube containing lithium heparin as anticoagulant (Vacutainer, BD) for the cell proliferation test and cytokine.

### Cell isolation and culture

The PBMCs were prepared from heparinized blood by Ficoll-Hypaque density-gradient centrifugation. To study the production of IFN-γ, IL-17 and IL-4, PBMCs were stimulated with PHA at a density of 2 × 10^6^ cells/ml. Isolated PBMCs were stimulated for 48 h and subsequently used for IFN-γ, IL-17 and IL-4 analysis by ELISA.

### ELISA for IFN-γ, IL-17 and IL-4

The protein levels of IFN-γ, IL-17 and IL-4 in the collected supernatants were measured using a Duoset ELISA development kit (R&D Systems). The minimal detectable concentration was 15 pg/ml for IFN-γ, IL-17 and IL-4. All samples were measured in duplicate.

### Statistical analysis

All analyses were performed using the Statistical Package for the Social Sciences statistical software for Windows, version 17.0 (SPSS Inc.). Group differences among diabetics and controls were analyzed using one-way ANOVA or nonparametric Kruskal–Wallis tests, depending on normality assumptions and the homogeneity of variances. The parameters showing statistically significant differences among all groups were further analyzed using the Mann–Whitney-U test or Student’s t-test. Differences within groups before and after stimulation were analyzed by Wilcoxon test. The correlations among study parameters were analyzed by Spearman’s correlation test. Differences in baseline gender among cases and controls were assessed using χ^2^ tests for proportions. For all tests, *P* values < 0.05 were considered statistically significant. Graphs were prepared by using Prism version 5 (GraphPad Software Inc., La Jolla, USA).

## Results

### Clinical characteristics of the patients

The clinical characteristics of the enrolled patients are shown in Table 1. With regard to age, sex, and body mass index (BMI) distribution, no significant difference was observed between the study groups (*P* = 0.945, *P* = 0.966, *P* = 0.177, respectively) (Table 1). Six patients were hypertensive in nAMD group and 7 patients were hypertensive in PCV. Twenty-five eyes had drusen in nAMD and only 6 eyes in PCV. Eight eyes had a pigment epithelium detachment (PED) in nAMD and 17 eyes in PCV. Subretinal fibrosis was present at a variable severity in 8 eyes in nAMD and 3 eyes in PCV.

**Table 1:** Clinical and biochemical characteristics of PCV, nAMD and control subjects

	Control subjects	PCV	nAMD	*P*
Number	18	33	27	0.945
Sex (m/f)	12/8	17/12	12/8	0.945
Age (years)	64.38 ± 11.92	64.79 ± 9.35	64.15 ± 9.30	0.966
BMI (kg/m^2^)	23.17 ± 2.02	21.21 ± 2.20	23.21 ± 3.64	0.177

*BMI* Body mass index, *PCV* polypoidal choroidal vasculopathy, *nAMD* neovascular age-related macular degeneration

Data are expressed as mean ± SD

### Concentrations of IFN-γ,IL-17A and IL-4 in the supernatants of cultured PBMCs

The results showed that IFN-γ and IL-4 was undetectable in the supernatants of unstimulated PBMCs of all subjects. A low concentration of IL-17A was detected in the supernatants of unstimulated PBMCs of patients with PCV and nAMD and control subjects, no significant difference was found among three groups (*P* = 0.436) (Table 2).

**Table 2:** Concentrations of IL-17A in culture supernatants of unstimulated or activated PBMCs from PCV, nAMD and healthy controls

	PBMCs	Control subjects	PCV	nAMD	*p*
		(*N* = 18)	(*N* = 33)	(*N* = 27)	
IL-17A	(unstimulated)	20.61 ± 45.23	45.25 ± 65.76	46.01 ± 65.76	0.436
	(stimulated)	1215.53 ± 875.21	1218.08 ± 860.30	942.09 ± 570.23	0.335
	Z	-3.724	-3.621	-2.934	
	P	0.000	0.000	0.003	

*PCV* polypoidal choroidal vasculopathy, *nAMD* neovascular age-related macular degeneration

All cytokines expressed as pg/ml. Data are means ± SD

After stimulation with PHA, IFN-γ, IL-17A and IL-4 production were significantly increased. The concentration of IFN-γ was 341.37 ± 341.96 pg/ml in PCV PBMCs samples and 393.42 ± 404.35 pg/ml in nAMD PBMCs samples. The concentration of IL-17A was 1218.08 ± 860.30 pg/ml in PCV PBMCs samples and 942.09 ± 570.23 pg/ml in nAMD. At the same time, the concentration of IL-4 was 42.08 ± 83.97 pg/ml in PCV PBMCs samples and 29.53 ± 69.46 pg/ml in nAMD PBMCs samples (Table 3).

**Table 3:** Concentrations of IFN-γ, IL-17A and IL-4 in culture supernatants of activated PBMCs from PCV, nAMD and healthy controls

	Control subjects	PCV	nAMD	*p*
	(*N* = 18)	(*N* = 33)	(*N* = 27)	
IFN-γ	135.90 ± 130.61	341.37 ± 341.96	393.42 ± 404.35	0.037
IL-17A	1215.53 ± 875.21	1218.08 ± 860.30	942.09 ± 570.23	0.335
IL-4	7.34 ± 23.24	42.08 ± 83.97	29.53 ± 69.46	0.012

*PCV* polypoidal choroidal vasculopathy, *nAMD* neovascular age-related macular degeneration

All cytokines expressed as pg/ml. Data are means ± SD

### IFN-γ and IL-4 levels were markedly elevated in the PCV and nAMD

**Fig. 1:**
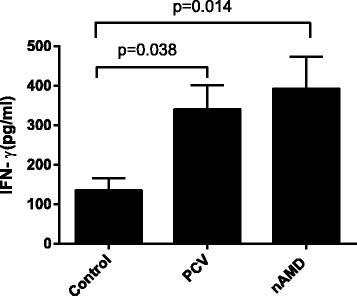
IFN-γ levels in the supernatants of cultured PBMCs measured by ELISA. Separated PBMCs were cultured with PHA (0.02 %) for 48 h. Data are represented as means ± SME

**Fig. 2:**
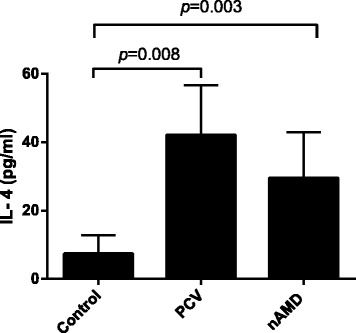
IL-4 levels in the supernatants of cultured PBMCs measured by ELISA. Separated PBMCs were cultured with PHA (0.02 %) for 48 h. Data are represented as means ± SME

The IFN-**γ** levels in PHA-stimulated cultures were higher in PCV and nAMD patients than that in healthy controls (*P* = 0.038,*P* = 0.014), while no difference was found between PCV and nAMD (*P* = 0.556) (Fig. 1). The IL-4 levels in PHA-stimulated cultures were higher in PCV and nAMD patients than that in healthy controls (*P* = 0.008, *P* = 0.003), while no difference was found between PCV and nAMD (*P* = 0.981). (Fig. 2). No significant difference in IL-17A levels in PHA-stimulated cultures were found among PCV, nAMD and control groups (*P* = 0.335) (Table 3).

### Correlation analyses in PCV and nAMD

**Fig. 3:**
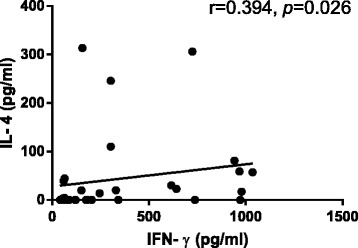
Correlation between IFN-γ and IL-4 in the supernatants of active PBMCs from PCV groups. The Spearman correlation test was used (*P* < 0.05 significant; *r* = correlation coefficient)

**Fig. 4:**
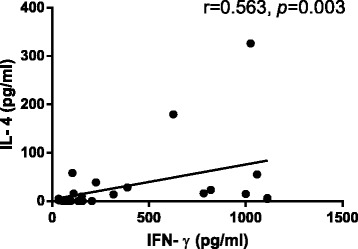
Correlation between IFN-γ and IL-4 in the supernatants of active PBMCs from nAMD groups. The Spearman correlation test was used (*P* < 0.05 significant; *r* = correlation coefficient)

The IFN-**γ** concentration was positively correlated with IL-4 concentration in activated PBMCs both in PCV and nAMD group (*r* = 0.394, *P* = 0.026 and *r* = 0.563, *P* = 0.003, respectively; Figs. 3 and 4). We found no correlation between IL-17A and IFN-γ concentrations in PCV and nAMD group (*r* = 0.051, *P* = 0.782; *r* = -0.092, *P* = 0.661).

## Discussion

There is more and more evidence for immune response involve in the pathogenesis and progression of AMD. Retinal pigment epithelial (RPE) cell is one of the most important systems that regulate the immune response in the eye. RPE cell represents a primary site of pathology in AMD and their function and phenotype may modify by cytokines and inflammatory mediators. Dysfunction of RPE cells could trigger the inflammatory response, which then could trigger the photoreceptor degeneration and neovascularization in the retinas [22]. This might be one of the mechanisms of PCV and nAMD development. A few data on these immuno-inflammatory cytokines expression in PCV were reported. We therefore examined the levels of Th cytokines of PCV and nAMD patients. Our result showed that IFN-γ levels in the supernatants of cultured PBMCs in PCV and nAMD patients were markedly elevated compared with normal controls, indicating that IFN-γ related inflammatory pathway may involve in the pathogenesis of PCV and nAMD.

Recent studies point towards an emerging relationship between IFN-γ and mechanisms underlying the pathogenesis of AMD. The interaction of pathways activated by IFN-γ is complex and not fully understood. IFN-γ is classically considered as a pro-inflammatory factor, yet in recent years, multiple studies find IFN-γ to mediate an immune-modulatory and protective function. IFN-γ increases complement factor H (CFH) expression in cultured human RPE [23]. CFH is a major inhibitor of the alternative complement activation pathway that plays a critical role in driving the inflammatory responses in outer retina and is associated with AMD pathogenesis [24]. In human endothelial cells IFN-γ inhibits the angiogenic activity of VEGF through activation of STAT1 pathway. This may inhibit excess angiogenesis process in wet AMD [25]. Another study suggests that IFN-γ induces VEGF expression in human retinal pigment epithelial cells [17]. The activation of the PI-3 K/mTOR/translational pathway is important for IFN-γ-mediated VEGF expression in RPE cells. It is known that STAT1-deficient mouse are highly susceptible to autoimmune disorders, thus STAT1 activation by IFN-γ may be important in mitigating AMD progression [13].

IFN-γ may also play a beneficial role by regulating Th17 cells. IL-17A is an important cytokine implicated in the pathogenesis of autoimmune disease [26]. Our data indicate that no significant difference in IL-17A levels in PHA-stimulated cultures among PCV, nAMD and control groups. It is reported that in vitro stimulated PBMCs carrying rs2275913AA genotype produced significantly more IL-17 than those with the GG genotype, and similar data were observed in both controls and AMD patients [11]. In paraffin-embedded tissues, expression of IL17A mRNA and the receptor IL17RC mRNA are higher in the macula of AMD patients than in control [27]. In vitro, IL17A induces RPE cell death characterized by the accumulation of cytoplasmic lipids and autophagosomes with subsequent activation of pro-apoptotic Caspase-3 and Caspase-9 [27]. Aqueous humor levels of IL-17 in eyes with nAMD or PCV are not statistically different from those in control eyes [28]. This result is consistent with our results. The different results are owing to different sample tissue, target population, detection mean and so on. Further studies are needed to clarify the role of Th17 in these patients.

In our analyses of serum cytokine profiles, IL-4 level in PHA-stimulated cultures was higher in PCV and nAMD patients than that in healthy controls. IL-4, a cytokine of Th2 subtype, plays an important role in humoral and cell-mediated immunity [29]. AGEs are a constituent of drusen and accumulate in Bruch’s membrane with age. IL-4 in supernatant samples from AGE stimulation of human RPE cells is 5.5 fold changes over controls [30]. Aqueous humor levels of IL-4 in eyes with nAMD or PCV are not statistically different from those in control eyes [28]. The levels of IL-4 in the aqueous humor samples from PCV patients are significantly higher than in the controls.

However, we were unable to distinguish nAMD and PCV from PHA-stimulated PBMCs based the expression of IFN-γ and IL-4. The reason remains unclear as the number of subjects enrolled in our present study was limited. One possibility is that IFN-γ and IL-4 is upregulated in both PCV and nAMD conditions but with different level and time window which may further trigger different signaling pathways and lead to different abnormal angiogenesis process. Another possibility is that PCV may have similar immune character with nAMD. PCV has many similarities with neovascular AMD, including clinical manifestations, genetic background, and epidemiological features [28]. It remains controversial as to whether or not PCV represents a subtype of nAMD. In clinical, some patients have possibility having CNV we cannot distinguish PCV from neovascular AMD. ‘Probable’ cases were determined if at least one of the following criteria were met: 1. only an abnormal vascular network is seen in ICGA. 2. Recurrent hemorrhagic and/or serous detachments of the RPE were observed [2]. From view of immune character, aqueous humor concentrations of CRP and IP-10 were elevated in eyes with PCV or nAMD. None of the 14 cytokines, including VEGF, IL-1α, IL-2, IL-4, IL-6, IL-8, IL-10, IL-12, IL-13, IL-15, IL-17, monocyte chemoattractant protein 1, were significantly different between eyes with nAMD and those with PCV [28]. It is reported that PCV and nAMD have common associations with the complement factor H (CFH) and the HtrA serine peptidase 1 (HTRA1) genes [31, 32], which suggests some genetic similarity between these two entities. ARMS2/HTRA1 locus has a strong and consistent association with both exudative AMD and PCV, suggesting the two disorders share, at least partially, similar molecular mechanisms [33].

In our previous study, we identified a variant in chromosome 9p21, well known to be associated with coronary artery disease and type 2 diabetes, was significantly associated with PCV but not with neovascular AMD in the Chinese Han population [34]. The rs42524 polymorphism is a risk allele for nAMD in a Han Chinese population [35]. However, rs42524 in COL1A2 confers different levels of susceptibility to nAMD and PCV [35] Thus, the different role of IFN-γ and IL-4 in the development of PCV and nAMD needs to be verified in further studies.

The major limitation of this study is the relatively small sample size. Additionally, no mechanistic and functional evaluations were conducted in this study. Extended cohorts with both PCV and nAMD patients offering higher statistical power will be necessary to confirm our results.

## Conclusion

In summary, our data show that circulating IFN-γ-producing Th1 cells and IL-4-producing Th2 cells is associated with the pathogenesis of PCV and nAMD. There is no significant difference in IFN-γ, IL-17A and IL-4 levels in the supernatants of cultured PBMCs between PCV and nAMD, indicating PCV may have the similar immune character with nAMD.

## Abbreviations

AMD, age-related macular degeneration; CFH, complement factor H; CNV, choroidal neovascularization; IL, interleukin; INF-γ, interferon-gamma; PBMCs, peripheral blood mononuclear cells; PCV, polypoidal choroidal vasculopath; RPE, retinal pigment epithelial; VEGF, vascular endothelial growth factor.
